# Patient outcomes following carotid endarterectomy are not adversely affected by surgical trainees’ operative involvement: A retrospective cohort study

**DOI:** 10.1016/j.amsu.2019.01.001

**Published:** 2019-01-21

**Authors:** Leo R. Brown, Jamie Anderson, Vish Bhattacharya

**Affiliations:** Department of Vascular Surgery, Queen Elizabeth Hospital, Gateshead, NE9 6SX, United Kingdom

**Keywords:** Carotid endarterectomy, Vascular surgery, Trainee, Education

## Abstract

**Background:**

Surgical training is an increasingly controversial topic. Concerns have been raised about both training opportunities becoming scarcer and poorer outcomes in operations led by surgical trainees; despite the evidence base for this being mixed. This retrospective cohort study aims to compare outcomes following carotid endarterectomy in patients who were operated on by a surgical trainee to those operated on by consultants.

**Materials and methods:**

Consecutive patients, who underwent carotid endarterectomy between 01/06/2012 and 1/12/2016, were entered into a prospectively maintained database. Patients were grouped according to whether a consultant or trainee vascular surgeon was the lead operating surgeon. Outcomes were 30-day mortality, 30-day stroke rate, operation time and complication rate.

**Results:**

One-hundred-and-twenty-one patients, with a mean age of 70.3 years, underwent carotid endarterectomy over a 4.5-year period. They were classified by the grade of the lead operating surgeon: consultant (n = 74) or registrar (n = 47). The median operative time was 117 min for consultants and 115 min for registrars with no significant difference between the two groups (p = 0.78). Three patients died in the post-op period, 2 secondary to post-operative stroke and a further 5 had nonfatal strokes. Grade of surgeon was also found to have no impact on 30- day mortality (p = 0.99) or stroke rate (p = 0.99). Sixty-six patients experienced post-operative complications, of varying severity, but no significant difference (p = 0.66) was found in incidence between trainee (57%) and consultant (53%) groups.

**Conclusion:**

Trainee involvement in carotid endarterectomy, with consultant supervision, leads to equivalent outcomes and represents a safe and useful training opportunity.

## Highlights

• There is a paucity of contemporary research assessing the safety of trainee involvement in carotid endarterectomy.
• Complication rates were higher for trainees but major complications were more common with consultant led operations.
• 30-day mortality is slightly higher in patients operated on by a consultant but not significantly so.
• Carotid endarterectomy can represent a safe and useful training opportunity for an appropriately supervised trainee.

## 1 Introduction

Surgical training is an increasingly controversial topic. The need to teach trainees, while simultaneously providing high quality, safe levels of care, is an increasingly challenging task. The days of ‘see one, do one, teach one’ are over and patient safety and adequate supervision are of progressively more focus. Significant concerns have been raised, however, that there has been a reduction in technical opportunities. Many fear that this could be having a detrimental impact on surgical training. Worldwide it is postulated that changes in practice may lead to prolonged time in training and, crucially, less technically skilled trainees [1,2].

While several factors have been identified as proponents, a key concern in the United Kingdom has been the implementation of the European Working Time Directive. While obvious patient safety benefits can be seen from limiting hours, the movement towards shift work, and the necessary extra rest days, are thought to have led to a reduction in opportunities for development of operative skills [[2], [3], [4], [5]]. This is not exclusive to the UK and is affecting surgical training internationally [6,7]. Alongside this, the centralisation of subspecialities, such as vascular surgery, has restricted many trainees’ experiences of these to teaching hospital rotations [8]. These issues are further exacerbated by efforts to increase the efficiency within pressured health budgets. Service provision is often prioritised leading to reduced operative opportunities for trainees [1]. Recommendations have been made for the formalisation of named operating lists for trainees [1,3] in attempt to separate and protect training opportunities, but these are far from common place. These concerns are powering a movement towards the development of more efficient, competency-based training programmes. While volume is of course important, this presents an opportunity to improve the quality of operative training experiences. It is important to note, however, that a recent systematic review concludes that there is insufficient evidence of good quality, to comment on the effect of reduced hours on surgical training [9]. With such extensive changes having occurred within surgical training, careful evaluation of practice is required of both efficacy and safety of current practice within surgical training.

The evidence surrounding the potentially detrimental effects of trainee involvement is very mixed. Some studies have found poorer outcomes for trainee involvement are demonstrated both in the emergency and elective settings in some circumstances [[10], [11], [12]]. Conversely, a number of papers have found no significant difference in major outcomes when looking into individual procedures, including Ivor Lewis oesophagectomy [13], hepatic and pancreatic resection [14], total hip arthroplasty [15] and strabismus surgery [16]. Perhaps then, it is best to narrow the spectrum of measurement and consider whether individual procedures represent safe and beneficial training opportunities.

There is a paucity of contemporary research into the safety of trainee involvement in carotid endarterectomy. This study aims to compare mortality and morbidity between consultant and UK surgical trainees, from a single centre in the North East of England, over a four and a half year period.

## 2 Material and methods

### 2.1 Patient population

This study describes a cohort of consecutive patients who underwent carotid endarterectomy between 01/06/2012 and 01/12/2016. All patients were treated at the author's institution. Inclusion criteria were all patients, over the age of 18, undergoing carotid endarterectomy for any reason. Bovine pericardial patch angioplasty was used in all cases.

### 2.2 Methods

A vascular nurse specialist entered patient demographics into a database at the study centre. Further data, including operation details and times, comorbidities, complication and mortality rates, was collected retrospectively by the authors from the hospital's electronic patient and operative record systems. Complications were graded using the Clavien-Dindo classification [17]. Stenosis and plaque morphology data were collected from carotid ultrasound, magnetic resonance angiography and CT reports as available.

Patients were grouped according to whether a consultant or trainee was the lead operating surgeon. The lead surgeon was identified as whoever performed the key components of the operation, as defined by the relevant Intercollegiate Surgical Curriculum Programme (ISCP) ‘Procedure Based Assessment’. Outcomes of interest were 30-day mortality, 30-day stroke rate, operation time and overall complication rate. This work has been reported in line with the STROCSS criteria [18] and registered in a publically accessible database (UIN: researchregistry4374).

### 2.3 Statistics

All data was collected and handled using a standardised spreadsheet (Excel 2010; Microsoft, Redmond, Washington, USA) and analyses were undertaken using IBM SPSS Statistics V23 software (SPSS, Chicago, Illinois, USA). Results are presented in numbers and percentages for the categorical variables and as mean with 95 per cent confidence interval for continuous variables. Median values with interquartile ranges were used for nonparametric data. Differences between groups were tested using Pearson's χ^2^ test for trend for categorical variables, and differences between continuous variables were analysed using ANOVA. Statistical significance was defined as P < 0.050 for all analyses.

### 2.4 Ethics

NHS Research and Ethics Committee approval was not required for this project as a retrospective analysis of data that had been collected for other purposes. Local approval was gained at the author's institute.

## 3 Results

### 3.1 Patient cohort

A consecutive series of one-hundred-and-twenty-one (n = 121) patients (76 men and 45 women) underwent carotid endarterectomy over a 4 ½ year period between June 2012 and December 2016. The mean age at the time of operation was 70.4 ± 9.4 (range, 44.8–87.8) years. All patients were admitted electively at the author's institution.

Patients were grouped according to whether a consultant or trainee vascular surgeon was the lead operating surgeon (Table 1). Three consultants and eight surgical trainees were recorded as being the lead operators across this cohort. Trainees were all surgical registrars at ST3 level and above or surgeons, not in a formal training programme, working at an equivalent level. In all operations where a trainee was the lead surgeon, a consultant was present in theatre supervising. The number of cases varied between trainees from 1 to 18. The median number completed per trainee was 3. A consultant was the lead operator in a slight majority (61%) of cases. The majority of cases were performed under general anaesthetic, in both consultant (58%) and trainee groups (66%), with no significant difference identified (p = 0.388). The remainder were performed under local anaesthetic.

**Table 1:** Patient demographics.

	Consultant-Led	Trainee-Led	*p-value*
Number of patients	61% (74)	39% (47)	
Age	70.5 (68.2, 72.7)	70.1 (67.5, 72.7)	0.811 ‡
ASA grade	2.8 (2.7, 2.9)	2.9 (2.8, 3.0)	0.483
Sex			0.365
Male	58% (43)	70% (33)	
Female	42% (31)	30% (14)	
Smoking status			0.068
Current	46% (34)	51% (24)	
Previous	15% (11)	28% (13)	
Non-smoker	39% (29)	21% (10)	
Comorbidities			
Diabetes Mellitus	20% (15)	21% (10)	0.894
Hypertension	82% (61)	87% (41)	0.479
Hyperlipidaemia	46% (34)	32% (25)	0.125
Ischaemic Heart Disease	35% (26)	34% (16)	0.902
Pulmonary Disease	15% (11)	19% (9)	0.536
Chronic Kidney Disease	20% (15)	19% (9)	0.880
Clinical Presentation			0.171
Stroke	53% (39)	30% (22)	
TIA	42% (31)	34% (25)	
Amaurosis Fugax	5% (4)	0% (0)	
Carotid Lesion Description			0.402
Extensive calcification	48% (36)	40% (19)	
Mixed calcification	18% (13)	28% (13)	
Fibrous Plaque	34% (25)	32% (15)	
Contralateral Carotid Artery			0.237
<60% stenosis	70% (52)	85% (40)	
60–80% stenosis	15% (11)	11% (5)	
>80% stenosis	8% (6)	2% (1)	
Complete occlusion	7% (5)	2% (1)	

Values in parentheses are percentages unless indicated otherwise; *values are mean (95 per cent C.I.). χ^2^ test for difference, except ‡ANOVA. Statistically significant values are highlighted in bold.

Age and ASA grade were comparable between groups. More men underwent the procedure overall and there was a further, not statistically significant (p = 0.365), predominance of males in the trainee group. Fewer patients smoked within the consultant-led group (p = 0.068) but more presented following stroke, though this was not statistically significant (p = 0.171). No difference was observed in the morphology of carotid lesions between groups (p = 0.402). Contralateral carotid artery stenosis was similarly comparable for consultants and trainees.

### 3.2 Overall outcomes

Overall 30-day mortality amongst the cohort was 2.5% (n = 3). The mean follow up was 3.40 (3.14, 3.65) years.

Sixty-five patients (53.7%) experienced post-operative complications. These varied greatly in severity, from temporary numbness around the wound site to major stroke. Complications were grouped according to the Clavien-Dindo grading system, that categorises based on severity from one to five. Twenty-eight patients (23.1%) experienced significant post-operative morbidity (Clavien-Dindo III to V) requiring further surgical intervention or resulting in failure or one or more organs.

### 3.3 Consultant vs. trainee

**Fig. 1:**
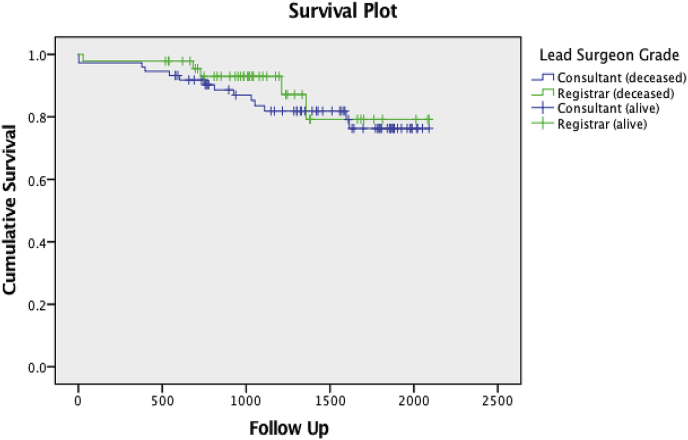
Kaplan meier survival curve (overall mortality across follow up period).

The consultant group was found to have a 30-day mortality of 2.7% (n = 2), slightly higher than 2.1% (n = 1) amongst the trainees (Fig. 1) but with no significant difference demonstrated between the groups (p = 0.66). All three patients died as a result of post-operative stroke. In one case this was preceded by a large haematoma causing airway obstruction and requiring an emergency return to theatre. A further 18.9% of the consultant group (n = 14) and 10.6% (n = 5) of the registrar group died during the follow up period (Fig. 1).

**Fig. 2:**
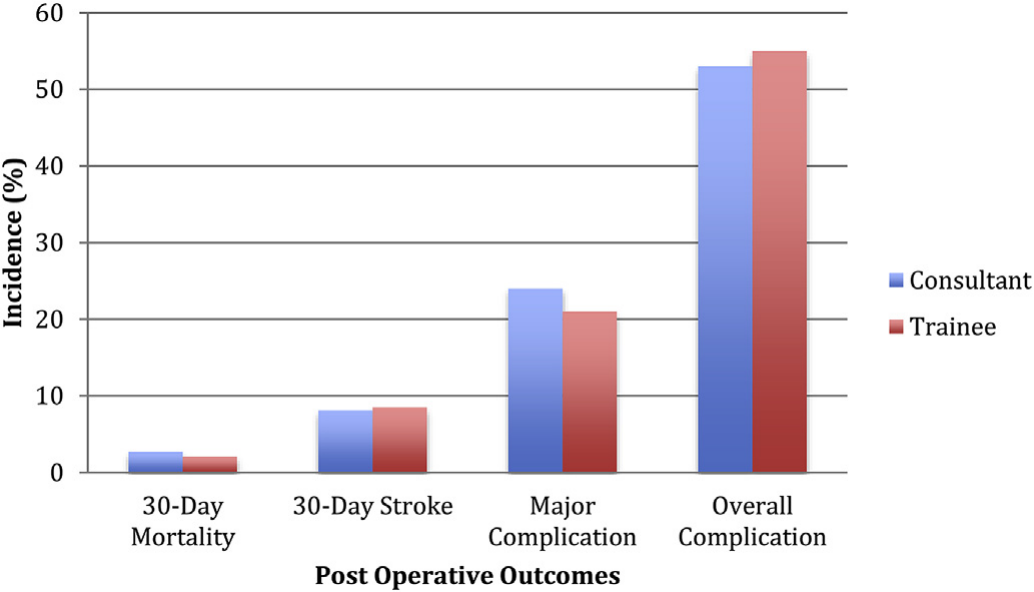
Patient outcomes by grade of lead surgeon.

Seven patients (5.8%) were diagnosed as having had a stroke during the 30-day postoperative period with a further three (2.5%) diagnosed as TIA. Overall incidence was very similar when comparing consultants (8.1%) and trainees (8.5%) (Fig. 2).

There was no significant difference in complication rates between groups (p = 0.78) although slightly more frequent in the registrar group (55.3% vs. 52.7%). Amongst the consultant group, 24.3% (n = 18) patients experienced a major complication (Clavien-Dindo III to V) compared with 21.3% (n = 10) in the registrar group with no significant difference between the two (p = 0.70).

Overall, operating time was recorded for 99.2% of cases. Mean operating time was 3 min shorter at 116 min (108, 123) when performed by a trainee compared with 119 (112, 125) minutes for consultants. This was not statistically significant (p = 0.78). Median length of stay was also slightly longer at 5 days [3,13] compared with 4 days (3, 6.5) for trainees but not significantly so (p = 0.12).

## 4 Discussion

This study found that trainee-led operating in carotid endarterectomy does not lead to worse patient outcomes or increase mortality. Ensuring good and equivalent trainee outcomes is vital to ensuring that the surgical training provided within our centres is of good quality and patient safety is not being compromised. With such extensive changes to working practice and training, the value of historic data is limited. This study looked at a contemporary patient cohort and as such was representative of the outcomes for modern UK surgical trainees.

A small number of studies have considered trainee involvement within carotid endarterectomy. Bradbury et al. identified the procedure to be a safe supervised training opportunity in the 1990s. Prior to this decade, they found carotid endarterectomy had been almost entirely consultant led. Despite a large increase in trainee led operations, they found no significant difference in outcomes [19]. A paper by Rijbroek et al., shortly afterwards, also found no difference in mortality or neurological morbidity, but did reveal significant patent selection, with consultants more likely to operate on asymptomatic patients [20]. In 2012, a large American study looked at over 25,000 carotid endarterectomies. They found that resident involvement, in any capacity including assisting, did not impact on complication rates [21]. Work from Germany and Italy has looked at trainee led, consultant supervised carotid endarterectomies. Both confirmed the there was no significant difference in major outcomes, such mortality and post-operative stroke, but found significantly longer operating times for trainees One study also revealed a greater rate of peripheral nerve damage as a complication in the trainee group [22,23].

Both mortality and incidence of post-operative stroke were similar to those within some of the existing literature [19,20] though slightly higher than those found by Lutz et al. and Cacioppa et al. [22,23]. Across all of the existing literature, outcomes have remained similar between consultants and supervised trainees [[19], [20], [21], [22], [23]].

Like most similar studies, patient numbers are limited owing to the small numbers of these operations being performed at individual centres. Future research might look to draw multi-centre data from a wider geographical area to more accurately assess outcomes. The study's retrospective design meant that data collection was reliant upon accurate documentation within patient records. Another limitation of this study is the potential selection bias that may exist with the decision of whether consultant or trainee performs the operation. While baseline demographics appeared comparable between the two groups, it is possible that consultants preferentially undertook particularly high-risk cases. No formal selection criteria were in place and future work may look to address this bias in a prospective study.

Awareness of patient safety and outcomes is paramount within surgery and all branches of medicine. In all cases, there was appropriate consultant supervision, and this alongside potentially preferential case selection, has led to equivalent outcomes. This study acts to as an update to supplement the existing literature and confirm the on-going safety and efficacy of vascular surgical training.

## 5 Conclusion

These findings confirm that carotid endarterectomy can represent a safe and useful training opportunity for an appropriately supervised trainee. There is a lack of contemporary research into the safety of trainee performed carotid endarterectomy in the UK. Although limited in size, this study verifies the efficacy of current practice and provides a basis for a larger multi-centre study. Surgical training programmes must continue to develop alongside the ever-changing working environment.

## Ethical approval

Formal NHS REC ethical approval not required for this research as a retrospective analysis of data collected for other purposes (notably the National Vascular Registry) with no patient involvement or intervention.

Approval was agreed locally with Associate Medical Director at the author's institute.

## Sources of funding

No funding was received for this work.

## Credit author statement

**Leo R. Brown**: Conceptualisation, Methodology, Formal Analysis, Writing – Original Draft, Writing – Reviewing & Editing, Visualisation, Project Administration. **Jamie Anderson:** Investigation, Data Curation, Writing – Original Draft, Writing – Reviewing & Editing, Visualisation, **Vish Bhattacharya:** Conceptualisation, Writing – Reviewing & Editing, Supervision.

## Conflicts of interest

No conflicts of interest to declare.

## Research registry number

researchregistry4374.

## Guarantor

Mr Vish Bhattacharya, senior author and supervising consultant.

## Provenance and peer review

Not commissioned, externally peer reviewed.
